# A Mini Review: Phase Regulation for Molybdenum Dichalcogenide Nanomaterials

**DOI:** 10.3390/nano14110984

**Published:** 2024-06-06

**Authors:** Xiaosong Han, Zhihong Zhang, Rongming Wang

**Affiliations:** Beijing Key Laboratory for Magneto-Photoelectrical Composite and Interface Science, State Key Laboratory for Advanced Metals and Materials, School of Mathematics and Physics, University of Science and Technology Beijing, Beijing 100083, China; xiaosonghan@xs.ustb.edu.cn

**Keywords:** molybdenum dichalcogenides, phase regulation, electronic doping, mechanical shift

## Abstract

Atomically thin two-dimensional transition metal dichalcogenides (TMDCs) have been regarded as ideal and promising nanomaterials that bring broad application prospects in extensive fields due to their ultrathin layered structure, unique electronic band structure, and multiple spatial phase configurations. TMDCs with different phase structures exhibit great diversities in physical and chemical properties. By regulating the phase structure, their properties would be modified to broaden the application fields. In this mini review, focusing on the most widely concerned molybdenum dichalcogenides (MoX_2_: X = S, Se, Te), we summarized their phase structures and corresponding electronic properties. Particularly, the mechanisms of phase transformation are explained, and the common methods of phase regulation or phase stabilization strategies are systematically reviewed and discussed. We hope the review could provide guidance for the phase regulation of molybdenum dichalcogenides nanomaterials, and further promote their real industrial applications.

## 1. Introduction

**Figure 1:**
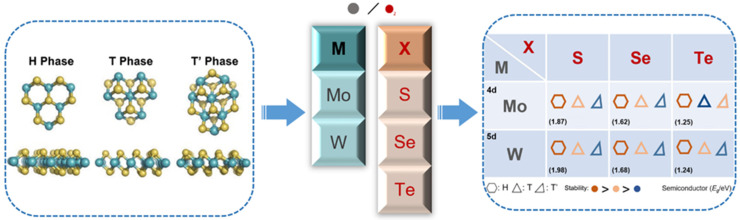
(Left box) Schematic diagrams for three common phase structures of the TMDCs, with the turquoise balls standing for M (Mo, W) and the yellow balls standing for X (S, Se, Te). (Right box) Summary of the phase stability and semiconductor properties, with the hexagon standing for the H phase, the regular triangle for the T phase, and the oblique triangle for the T′ phase.

Transition metal dichalcogenides (TMDCs) are typically layered materials, and mono- or few-layer two-dimensional (2D) TMDC nanomaterials can be obtained by various techniques [1,2], holding extraordinary and special characteristics [3,4,5,6], which have been widely studied in the field of electronics [7], optoelectronics [8,9], energy storage [10,11], catalysis et al. [12,13,14,15]. TMDCs have a general formula of MX_2_ (M stands for transition metal, and X stands for chalcogenide). The monolayer TMDC usually has a sandwich structure, where the M layer of transition metal atoms is sandwiched by two X layers of chalcogenide atoms, and the M and X atoms are combined by a strong covalent bond. For TMDCs, there exist several phase structures, basically including 1H/2H (1H for monolayer and 2H for multilayer) and 1T, where H and T represent hexagonal and trigonal, and the numbers indicate the number of layers in the unit cell, respectively [7]. More specifically, the 1H phase corresponds to a trigonal prismatic coordination of the M atoms, with two layers of X atoms vertically aligned, leading to an ABA stacking, while the 1T phase corresponds to an octahedral coordination of the M atoms, with one of the X layers shifted compared to the other leading to an ABC stacking [16,17]. Some TMDCs have a special twisted octahedral structure, which is named the 1T′ phase. Similar to the 1T phase, the 1T′ phase also shows an octahedral coordination but distorted so that, for example, there are two distances of neighboring Mo atoms of 2.77 Å and 3.88 Å in 1T′-MoS_2_, different from the same distance of 3.23 Å in 1T-MoS_2_ [18,19]. These three typical phase structures are shown in Figure 1. Also, we summarize the stability and bandgap of the semiconducting phase (monolayer) of the most widely concerned TMDCs (MX_2_: M = Mo, W; X = S, Se, Te). We can see that all of the concerned TMDC nanomaterials have a thermodynamically stable H phase, appearing in the semiconductor characteristics [20]. It is especially worthy of note that different stacking orders of monolayers may also lead to distinct phase structures of bi- or few-layer TMDCs, but this is not in the scope of this mini review, which only discusses the phase transformation caused by the lattice changes within the layer. Therefore, we will not go into too much detail here on that.

**Figure 2:**
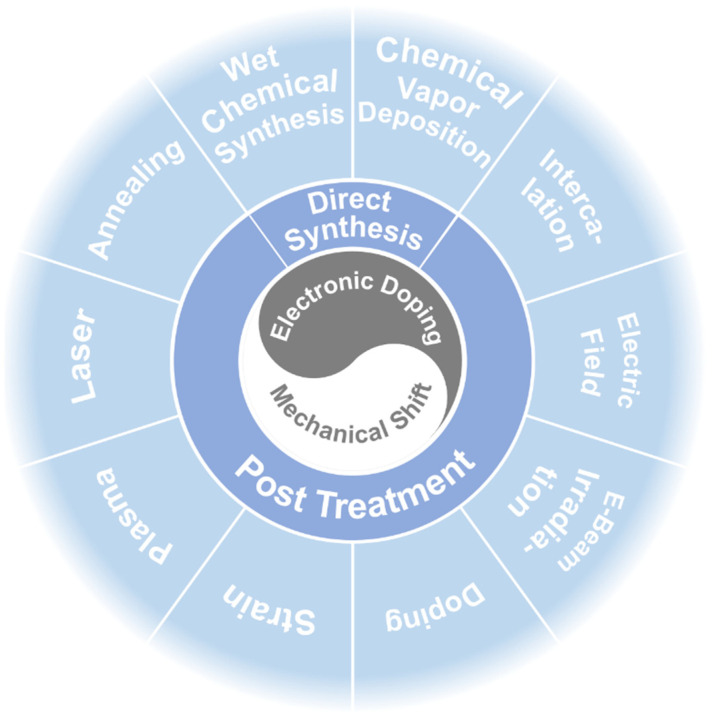
An overview of the phase regulation techniques for TMDC nanomaterials.

Different phase structures lead to huge disparity in the properties of the TMDC nanomaterials [21,22,23,24,25]. For example, molybdenum disulfide (MoS_2_) exhibits all three phase structures. Normally, monolayer MoS_2_ exists as a thermodynamically stable 1H phase, exhibiting the characteristics of a semiconductor with a band gap of 1.87 eV, while the 1T and 1T′ phases are metastable and present metallic characteristics [26,27,28,29,30]. These phase structures can be principally converted from each other by physical and chemical regulation techniques [31,32,33]. As the research of TMDC nanomaterials has gained more and more attention in recent years, most studies are focusing on the stable phase structure (H phase). On the other hand, TMDC nanomaterials with metastable phase structures also show unique and excellent performance, which should arouse the attention of researchers [34,35,36]. Chief of all, how to regulate and achieve controllable phase structures needs to be further studied, and the phase stabilization strategies for metastable TMDC nanomaterials should be cleared. Therefore, in this mini review, focusing on the most widely concerned molybdenum dichalcogenides (MoX_2_: X = S, Se, Te), we summarize their phase structure and stability and discuss the two generally accepted dominating mechanisms of the phase transformation, which could be realized through electronic doping and mechanical shift (Figure 2). Based on these mechanisms, various techniques, mainly direct synthesis methods, including chemical vapor deposition (CVD) and wet chemical synthesis, and post treatments, including ion intercalation, applying an electric field, e-beam irradiation, doping, applying strain, plasma bombardment, laser irradiation, and annealing treatment, have been discussed. The advantages, disadvantages, and application scopes of these techniques are also summarized.

## 2. Characterization Techniques for TMDC Phase Structure

Techniques commonly used for characterizing the phase structure of TMDC nanomaterials include optical microscopy (OM), Raman spectroscopy, X-ray diffraction (XRD), and transmission electron microscopy (TEM).

**Figure 3:**
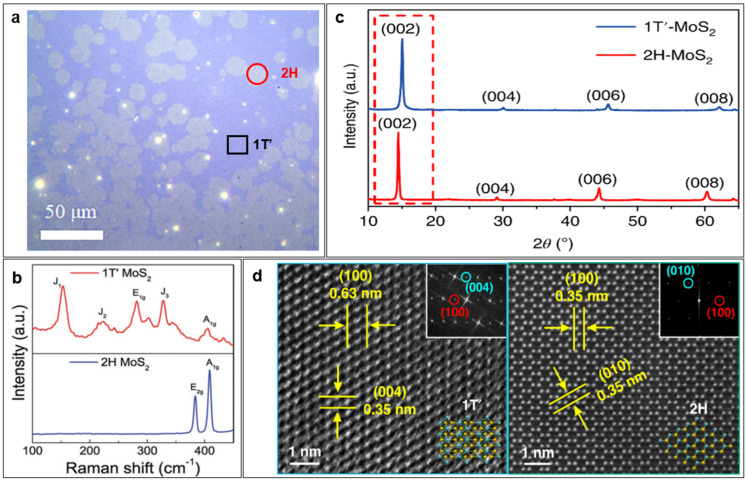
Common characterization techniques of phase structure of TMDC nanomaterials. (**a**) OM image of 1T′-2H MoTe_2_ homojunction. Reprinted with permission from Ref. [37]. 2022, AIP Publishing. (**b**) Raman spectra of 1T′-MoS_2_ and 2H-MoS_2_. Reprinted with permission from Ref. [38]. 2019, John Wiley & Sons. (**c**) XRD patterns of 1T′- and 2H-MoS_2_ crystals. Reprinted with permission from Ref. [39]. 2018, Springer Nature. (**d**) Z-contrast STEM images of 1T′- and 2H-MoTe_2_. Reprinted with permission from Ref. [40]. 2023, Springer Nature.

OM is the easiest and most rapid characterization technique to gain basic information on the morphology, thickness, distribution, etc. Also, it can be used to distinguish different phases if samples with different phases show distinct morphologies or thicknesses or have a large disparity in refraction indices. The most common example is to use OM to observe MoTe_2_ grown on a substrate, as shown in Figure 3a [37]. It can be inferred that the 2H-MoTe_2_ domains were inserted in the 1T′-MoTe_2_ film, forming a 1T′-2H MoTe_2_ homojunction.

The Raman shift could provide valuable insights into the molecular and lattice vibrations within the sample, enabling the determination of its chemical composition, crystal structure, molecular arrangement, as well as other properties. Raman spectroscopy offers a relatively convenient and accurate way to detect different phase structures of TMDC nanomaterials over micron-sized areas. For example, 1T′ and 2H MoS_2_ have distinct frequency shifts and can be easily differentiated from each other by their Raman spectra, as shown in Figure 3b [38].

XRD is a powerful experimental technique used to analyze crystal structures, and thus, it is often applied to characterize TMDC nanomaterials. Due to the characteristics of X-rays, XRD samples need to be powders or thick films. If the film is too thin, grazing incidence XRD (GIXRD) is needed. One example is shown in Figure 3c, where the peaks of 1T′-MoS_2_ are shifted higher than those of 2H-MoS_2_ [39].

TEM is a widely utilized high-resolution microscope for studying the microstructure of materials. For monolayer TMDCs, the 1H, 1T, and 1T′ phases can be unambiguously distinguished by TEM, while for multilayer ones, the 1T and 2H phases, displaying a three-fold symmetry in the *ab* plane, are virtually undistinguishable. For example, the Z-contrast high-resolution scanning TEM (HR-STEM) images in Figure 3d show a monoclinic lattice structure of 1T′-MoTe_2_ and a hexagonal atomic structure of 2H-MoTe_2_ [40].

## 3. Mechanisms of Phase Transformation

Due to the similar characteristics and structures, mechanisms of phase transformations for different MoX_2_ nanomaterials are parallel [41,42]. Here, we take monolayer MoS_2_ as an example to discuss the mechanism of phase transformation in detail.

**Figure 4:**
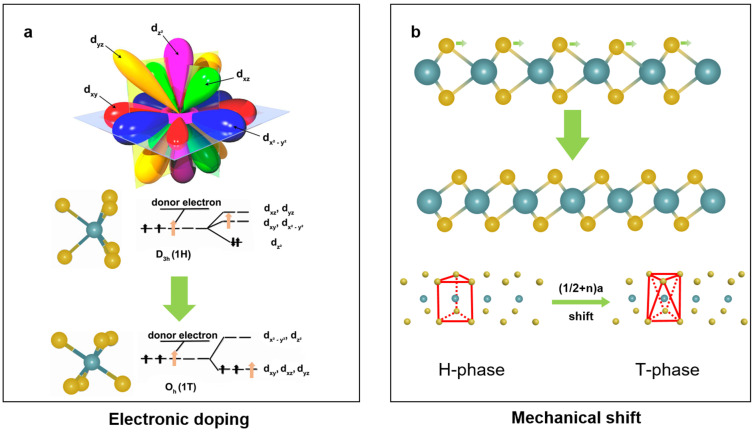
Schematic diagrams for two kinds of phase transition mechanisms. (**a**) Electronic doping mechanism. Reprinted with permission from Ref. [44]. 2011, American Chemical Society. (**b**) Mechanical shift mechanism.

Generally, there are two kinds of mechanisms to explain the phase transformation. The first one is proposed from the view of the regulation of electronic band structure. According to the classical electron hybrid orbital theory, for the 1H phase, the 4d-orbitals of the Mo atoms split into three groups, namely (1) d_z_^2^, (2) d_x_^2^_-y_^2^, d_xy_, and (3) d_xz_, d_yz_, while for the 1T phase, the 4d-orbitals split into two groups, namely (1) d_x_^2^_-y_^2^, d_z_^2^ and (2) d_xy_, d_xz_, d_yz_ [43]. The simplified representations of the above description are shown in Figure 4a, as well as the electron filling state [44]. Such electron occupation makes 1H-MoS_2_ stable and 1T-MoS_2_ unstable. On the other hand, when doping MoS_2_ with donor atoms, the additional electron occupation decreases the stability of 1H-MoS_2_ but increases the stability of 1T-MoS_2_. Therefore, based on the electronic doping mechanism, electrons doping to 1H-MoS_2_ directly cause a phase transition to the 1T phase [45]. The phase transformation mechanism between the 1H and 1T′ phases is similar. Initially, the 1T phase was discovered in the process of alkali metal (Li and K) intercalation of layered TMDC nanomaterials [46,47]. In 2018, Jiao and coworkers first directly synthesized the 1T′ MoS_2_ by the chemical vapor-deposited method following this mechanism [48]. Other methods, such as applied electric field [49], electron beam irradiation [50], and doping [51], can also realize the phase transformation from a stable phase to a metastable one.

The second mechanism is explained from the view of the regulation of the lattice structure. When one X layer is shifted by (1/2 + n)a (a = lattice constant), the 1H phase corresponding to a trigonal prismatic structure will transit to the 1T phase corresponding to an octahedral structure, as shown in Figure 4b [20]. This lattice shift is relatively easy to implement, e.g., the introduction of surface defects (like vacancies, foreign atoms), and there are many ways, such as applying strain [52], plasma bombardment [53], laser radiation [54], annealing treatment [55], to achieve this.

## 4. Phase Regulation Methods for TMDC Nanomaterials

According to the above discussion, phase transformation can occur mainly in two ways, electronic doping and mechanical shift. Many techniques can realize these two mechanisms, and basically, we divide them into two categories, direct synthesis [56] and post processing [57,58].

### 4.1. Phase Regulation of TMDC Nanomaterials by Direct Synthesis

CVD is an efficient and ideal means to prepare large-area, high-quality TMDC nanomaterials [59,60]. By using the CVD method, not only the morphology but also the phase structure of TMDC nanomaterials can be regulated [61,62]. The main way to achieve phase regulation in CVD is to optimize the precursor, substrate, or thermodynamic condition to realize the electron doping during the growth process or control the defect density or lattice strain to introduce lattice shift.

**Figure 5:**
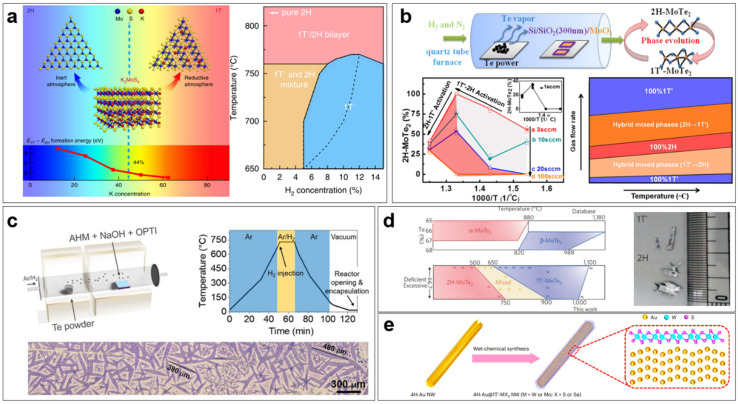
Regulating TMDs phase structure by direct synthesis. (**a**) Phase-selective growth of 1T′-MoS_2_ monolayers and 1T′/1H heterophase bilayers by CVD. Reprinted with permission from Ref. [48]. 2018, Springer Nature. (**b**) Phase regulation of MoTe_2_ by tellurization rate in CVD. Reprinted with permission from Ref. [65]. 2017, American Chemical Society. (**c**) CVD synthesis of large-size monolayer 1T′-MoTe_2_ single crystals by optimizing precursor distribution. Reprinted with permission from Ref. [66]. 2021, American Chemical Society. (**d**) Synthesis of bulk single crystals of 2H- and 1T′-MoTe_2_ using the flux method. Reprinted with permission from Ref. [55]. 2015, Springer Nature. (**e**) Schematic illustration of the quasi-epitaxial growth of 1T′-MoS_2_, MoSe_2_, WS_2_, and WSe_2_ monolayers on 4H-Au nanowires by wet-chemical synthesis. Reprinted with permission from Ref. [68]. 2022, Springer Nature.

Liu et al., for the first time, successfully achieved 1T′-MoS_2_ by using the specially designed precursor, K_2_MoS_4_, by the CVD method [48]. Furthermore, by applying reductive and inert atmospheres, they can tune the K^+^ concentration in the reaction product and the stability of the 1T′ phase and 1H phase, and thus realize the phase-selective growth of 1T′-MoS_2_ monolayers and 1T′/1H heterophase bilayers (Figure 5a). Specifically, the 1T′ phase becomes more stable than the 1H phase at a K^+^ concentration of >44%. Following their strategy, Wang et al. realized the controlled growth of 1T′-MoS_2_ nanoribbons on 1H-MoS_2_ nanosheet this year [45]. Besides the precursor, the substrate can also dope the TMDC and thus regulate its phase structure. Cheng et al. demonstrated the selective growth of 1H- and 1T-MoSe_2_ on pristine Au(111) and Se-pretreated Au(111), respectively [63]. Their theoretical calculation results show Mo atoms would be intercalated between the MoSe_2_ and Se-pretreated Au(111) during growth, and the Mo-covered Au(111) can transfer electrons to the MoSe_2_ monolayer and further stabilize its 1T′ phase.

The CVD growth of MoTe_2_ with a controlled phase structure has attracted wide attention. MoTe_2_ is a kind of special material; its 1T phase is the most unstable, and the free energy difference between the semiconducting 2H phase and metallic 1T′ phase is very small. Therefore, it is relatively easy for MoTe_2_ to reversibly transform from the 2H phase to the 1T′ phase [37]. On the other hand, it is challenging to synthesize pure-phase MoTe_2_ crystals. Zhou et al. realized the selective synthesis of pure 2H- and pure 1T′-MoTe_2_ films by choosing proper Mo precursors using the CVD method [64]. When using MoO_3_ as the Mo precursor, which reacts more easily with Te, pure 2H-MoTe_2_ can be achieved; when using metal Mo or MoO_x_ (x < 3) as the precursor, pure 1T′-MoTe_2_ can be achieved. The authors explained that the different results lie in the strain developed during the CVD process, and the small volume change from MoO_3_ to MoTe_2_ leads to the formation of 2H-MoTe_2_, which is more stable in the absence of strain. The strain in 2H-MoTe_2_ may lead to a lattice shift and, further, a phase transition to the 1T′ phase. Therefore, the above growth results can be explained by the mechanical shift mechanism. Chang et al. found that the tellurization rate is the key factor determining the phase structure of MoTe_2_, and the fast tellurization rate leads to the pure 1T′ phase [65]. They employed a two-heating zone CVD method and controlled the tellurization rate by adjusting the tellurization temperature and the carrier gas flow rate to realize the phase regulation of MoTe_2_ films (Figure 5b). The mechanism of such regulation is that the kinetics of tellurization would affect the strain relaxation during the CVD process and further the stability of different phase structures.

Although many works have realized the synthesis of pure-phase MoTe_2_ films, achieving large-area pure-phase MoTe_2_ monolayers is still a great challenge. Coletti et al. successfully prepared large monolayer 1T′-MoTe_2_ single crystals by using a liquid Mo precursor (ammonium heptamolybdate tetrahydrate, AHM), growth promoter (NaOH), and density gradient medium (commercial Opti Prep, OPTI), which makes the Mo precursor distribute uniformly over the entire substrate (Figure 5c) [66]. However, the monolayer 1T′-MoTe_2_ has poor stability. To decrease its degradation in environmental conditions, the authors developed a scalable BN encapsulation approach that can increase the lifetime of the monolayer 1T′-MoTe_2_ from a few minutes to more than a month.

Besides CVD, there are also some other unique synthesis techniques to realize the controllable regulation of TMDC nanomaterial phase structures. Keum et al. successfully prepared bulk 2H- and 1T′-MoTe_2_ single crystals using the flux method, where Mo and Te powders were completely dissolved in liquid NaCl during the crystal growth, effectively avoiding the Te deficiency [55]. In their experiment, 2H-MoTe_2_ is more stable at low temperatures, while 1T′-MoTe_2_ is more stable at high temperatures (Figure 5d). Additionally, the high-quality bulk 1T′-MoTe_2_ crystals are semimetals and exhibit a high carrier mobility of 4000 cm^2^ V^−1^ s^−1^, and few-layered ones are semiconductors with a bandgap of 60 meV. Wet-chemical synthesis is also a popular bottom-up approach to synthesizing nanomaterials. Li et al. realized the synthesis of high-purity and stable 1T′-MoS_2_, MoSe_2_, WS_2_, and WSe_2_ monolayers via the wet-chemical method using 4H-Au nanowires as templates (Figure 5e) [67]. The strong interaction between the template and TMDC monolayers, and charge doping from the solution and the template stabilize the 1T′ phase. Liu et al. successfully prepared nanosized 1T′-MoS_2_, MoSe_2_, WS_2_, and WSe_2_ monolayers by a colloidal synthesis method [68]. In their synthesis, the precursor of the high-concentrate monomer was hot-injected, and the nucleation occurred explosively, enabling the formation of metastable 1T′-phase crystals. They also used surface ligands to stabilize the metastable phase structure, preventing its transition to a stable one. Li et al. prepared monolayer TMDC/bismuth (Bi) core-shell nanoparticles via colloidal chemical synthesis and by etching away Bi-core high-purity 1T-MoS_2_, MoSe_2_, WS_2_, and WSe_2_ single-layer hollow spheres were achieved [36]. Their theoretical calculation and experimental characterization results demonstrate that the 1T phase is not a consequence of the Bi etching process but the strong interaction between TMDC monolayers and Bi cores and probably carrier transfer.

### 4.2. Phase Regulation Methods of TMDC Nanomaterials by Post Processing

In addition to the direct synthesis method, various post-processing techniques can also regulate the phase structures of TMDC nanomaterials. Based on phase regulation mechanisms, the techniques can be classified into two categories: ones introducing electronic doping and ones introducing mechanical shift.

#### 4.2.1. Post-Processing Techniques Inducing Electronic Doping

Intercalation is the earliest and most common method to realize the phase regulation of TMDC nanomaterials. The intercalation materials mainly include alkali metals (Li, Na, and K), and intercalation can be realized by sonication and electrochemical techniques [69,70,71,72,73,74,75,76,77,78].

Lithium is the earliest and most popular element of alkali metal intercalation in the study of TMDC nanomaterial phase regulation. In 1983, Py et al. first realized the transition from the 2H to 1T phase when chemically stripping MoS_2_ using lithium intercalation [46]. When lithium is inserted into bulk MoS_2_, the charge will be transferred from the lithium to MoS_2_, resulting in the conversion of the 2H phase to the 1T phase. Theoretical studies show that the octahedrally coordinated MoS_2_ (including the 1T and 1T′ phases) is more stable than the 1H phase when the excess charge density is above −8.33 × 10^14^ cm^−2^ in the stripped nanosheet sample [79]. At the same time, the intercalation of Li^+^ weakens the van der Waals interactions between the layers, resulting in the expanding layer spacing. Wang et al. further studied the layer distance expansion of vertically aligned MoS_2_ nanofilm (polycrystals with a grain size of about 10 nm) via the Li^+^ intercalation and gave the relationship between the Li intercalation potential, Li content x in Li_x_MoS_2_, and layer spacing [72].

**Figure 6:**
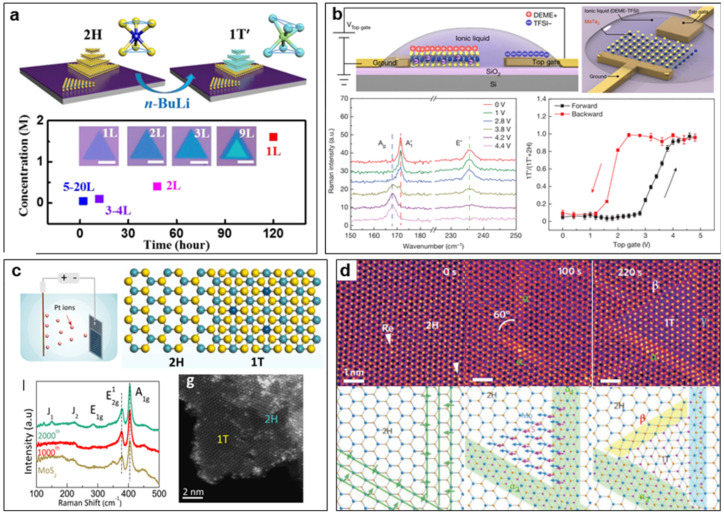
Post-processing techniques inducing electronic doping to regulate TMDC phase structure. (**a**) Li^+^ intercalation-induced phase transition of CVD-grown MoS_2_ flakes with different number of layers. Reprinted with permission from Ref. [74]. 2018, American Chemical Society. (**b**) Electrostatic gating-induced phase transition of MoTe_2_ between the 1H and 1T′ phase. Reprinted with permission from Ref. [49]. 2017, Springer Nature. (**c**) Pt doping-induced partial 2H to 1T phase conversion of MoS_2_. Reprinted with permission from Ref. [81]. 2021, Elsevier. (**d**) Dynamic phase transition process of electron irradiation-induced 1H to 1T phase transition in Re-doped MoS_2_ studied by in situ STEM. Reprinted with permission from Ref. [84]. 2014, Springer Nature.

Many works have found that the Li^+^-intercalated 1T phase can be spontaneously distorted to the 1T′ phase, which is believed to be a more stable structure in this condition [73,74,75]. Sun et al. studied the 2H to 1T′ phase transition of CVD MoS_2_ with a different number of layers by Li^+^ intercalation and found the injected electron concentration and treatment time required for the phase transition decrease with the increased thickness (Figure 6a) [74]. Due to its metastable nature, the 1T′-phase crystal may easily convert to a stable 2H phase, especially after air exposure, annealing, or aging. To stabilize the 1T′ phase, Tan et al. developed an effective phase engineering method, where Li atoms were intercalated into few-layer 2H-MoS_2_ and then thermally hydrogenated to form LiH, realizing the complete transformation of 2H-MoS_2_ to 1T′-MoS_2_ with long-term stability (>3 months) in the air, and this method is also widely applicable to other alkali metals and TMDC nanomaterials [75].

Applying an external electric field is also a feasible way to regulate the phase of TMDC nanomaterials. Based on density functional theory (DFT) calculation, Li et al. proposed that electrostatic gating can realize the 1H to 1T(1T′) phase transition in some monolayer TMDCs by applying a large enough gate voltage with proper dielectric [27]. Additionally, for MoTe_2,_ they found that the substitution of Mo atoms by W atoms to form the alloy Mo_x_W_1−x_Te_2_ could reduce the required voltage to realize the phase transition. In 2017, Wang et al. first reported the experimental demonstration of the phase transition of MoTe_2_ between the 1H and 1T′ phase by electrostatic gating [49]. They constructed an ionic liquid field-effect transistor to realize the electrostatic gating of MoTe_2_ and applied Raman spectroscopy to monitor the phase structure evolution in situ when sweeping the gate voltage (Figure 6b). The experimental results show that, as the gate bias is higher than 2.8 V, the transition from 1H to 1T′ phase begins, and the transition completes at the bias of 3.8 V. When the gate voltage is swept backward, the 1T′ phase converts to the 1H phase, but there is hysteresis. By extra optical characterization, they demonstrated that the phase transition would not change the lattice orientation of the MoTe_2_ crystal, and such electrostatic gating-induced structural phase transition occurs simultaneously across the whole sample. This electrostatic-gating regulation of phase structures opens new possibilities for developing phase-change devices based on atomically thin TMDC nanomaterials. Zhang et al. designed vertical 2H-MoTe_2_- and Mo_1−x_W_x_Te_2_-based resistive random access memory (RRAM) devices and demonstrated an electric-field-induced phase transition from the 2H to 1T phase in both of these two kinds of materials, accounting for reproducible resistive switching within 10 ns between a high resistive state and a low one of devices [80]. In particular, they observed a distorted transient structure during the phase transition from 2H to 1T phase.

Owing to the atomic thickness and the weak van der Waals force between the TMDC layers, foreign atoms can be easily doped into TMDC nanomaterials to regulate their phase structure. Dopants can be metallic or non-metallic elements. Liu et al. synthesized the Pt-doped MoS_2_ by a potential-cycling method, which introduces the Pt dopants into the MoS_2_ lattice (mainly Pt atoms substituting Mo atoms) and leads to a partial 2H to 1T phase transition of MoS_2_ (Figure 6c) [81]. The authors ascribed the phase transition to the electron doping caused by the introduction of foreign Pt atoms. Young et al. investigated the stability of chalcogen-alloyed MoTe_2_ (MoTe_2−x_X_x_) using the first principal DFT and proposed that chalcogen alloying is also a potential way to realize the reversible phase transition between 2H and 1T′, which can reduce the energy difference between 2H and 1T′ phase by both the electron doping and chemical strain [82]. Deng et al. realized the partial 2H to 1T phase transition by doping non-metallic N atoms in MoSe_2_ nanosheets by NH_3_ annealing and obtained a 1T-2H MoSe_2_ heterostructure [83]. The (semi)metallic-phase TMDC nanomaterials obtained by the doping method have higher stability and generally will not change back to the original semiconductor phase. However, some of the intrinsic excellent properties of TMDC nanomaterials may no longer be maintained, and the balance of pros and cons needs to be weighed circumstantially.

Electron irradiation is another way to realize the electron doping to TMDC nanomaterials to regulate the phase structure. Lin et al. studied the electron irradiation-induced phase transformation between 1H and 1T phases in single-layered Re-doped MoS_2_ at elevated temperatures (400 °C~700 °C) by in situ scanning transmission electron microscopy (STEM) [84]. They observed the dynamic process of the structural transition involving the slip of the atomic layer and the formation of two new phase boundaries (β and γ), as shown in Figure 6d. It should be noted that such phase transitions can only occur in the e-beam irradiation region, and this can be explained by the fact that the continuous e-beam irradiation may accumulate negative charges, together with the small amount of n-type Re doping, enabling high electron doping and triggering the phase transformation. Y. Katagiri et al. also used electron beam irradiation to induce the phase transition locally to achieve in-plane 1T-2H heterojunction [85]. E-beam irradiation can be used for very precise phase regulation and to construct heterostructures accurately. It should be noted here that electron beam irradiation may also induce the formation of vacancies and the spread of strain in the lattice, further leading to the 2H to 1T phase transition [86].

#### 4.2.2. Post-Processing Techniques Inducing Mechanical Shift

There is a great diversity of post-processing treatments that can induce mechanical shift in TMDC nanomaterials and regulate their phase structure, commonly including plasma treatment, applying strain, laser irradiation, and thermal annealing. The first main class of phase regulation is the traditional physical techniques—plasma treatment and strain treatment.

**Figure 7:**
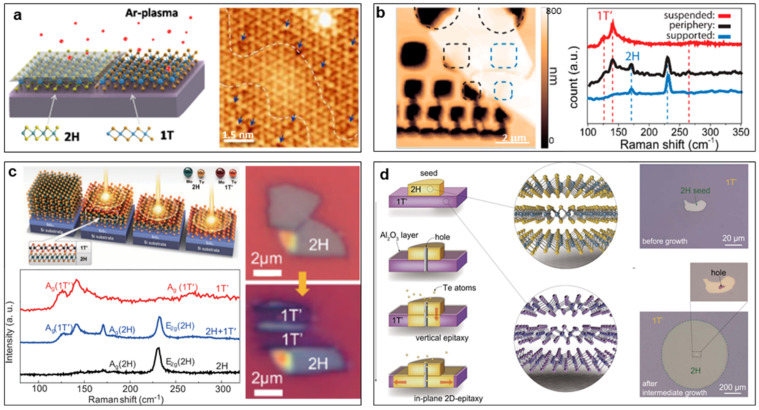
Post-processing techniques inducing mechanical shift to regulate TMDC phase structures. (**a**) Weak argon-plasma treatment-induced local phase transition from 2H to 1T of MoS_2_. Reprinted with permission from Ref. [53]. 2017, American Chemical Society. (**b**) Tensile strain of 0.2%-induced phase transition from 2H to 1T′ in MoTe_2_. Reprinted with permission from Ref. [90]. 2016, American Chemical Society. (**c**) Laser irradiation-induced local phase transition from 2H-MoTe_2_ to 1T′-MoTe_2_. Reprinted with permission from Ref. [54]. 2015, The American Association for the Advancement of Science. (**d**) Transformation of polycrystalline 1T′ MoTe_2_ wafer into single-crystal 2H-MoTe_2_ by thermal annealing in a Te atmosphere. Reprinted with permission from Ref. [92]. 2021, The American Association for the Advancement of Science.

Plasma treatment is a kind of dry treatment that is fast, effective, clean, and controllable. Different gas plasmas can be used to treat the TMDC nanomaterials, but careful control is needed to activate the phase transition by inducing the slip of the top chalcogen layer and avoiding damage to the sample. Zhu et al. achieved a local phase transition of MoS_2_ by weak argon-plasma treatment [53]. After the 40s plasma treatment, the 1T phase area can reach 40%, and the domain size of the 1T phase is several nanometers (Figure 7a). Using atomically resolved scanning tunneling spectroscopy (STM), they observed a few S-vacancies in the 1T phase, suggesting the S vacancies activate the phase transition and further stabilize the 1T structure. Such MoS_2_ samples with a high density of 1H-1T phase boundaries exhibit excellent hydrogen evolution reaction performance [87]. Nan et al. also realized the phase transition in the monolayer and few-layer MoTe_2_ by employing a soft hydrogen plasma treatment [88].

Applying strain is a very typical and intuitive method to introduce a mechanical shift into the TMDC lattice to regulate its phase structure. Several theoretical works have suggested that the vacancies or tensile strain could reduce the energy difference between the 2H and 1T′ phases, improving the possibility of the phase transition from 2H to 1T′ [27,52,89]. Strain can be applied by deforming flexible substrates using atomic force microscopy (AFM) and other standard experimental methods [89]. Song et al. experimentally demonstrated a tensile strain of 0.2%-induced phase transition from 2H to 1T′ in MoTe_2_, implemented by contacting the suspended MoTe_2_ sample with an AFM tip [90]. The Raman results show that the suspended region feature signals the 1T′ phase, while the supported region features signals the 2H phase, suggesting a phase transition from the 2H to 1T′ in the suspended strained region (Figure 7b). The effect of strain treatment can be eliminated by natural aging, annealing aging, and other methods.

Generally, energy acquisition is a necessary condition to modulate the material structure, so it is the most direct method to change the structure through laser irradiation or thermal annealing. Intuitively, laser irradiation could increase the energy of the lattice and thus accelerate the phase transition from a metastable phase to a stable one. Yu et al. observed such laser-induced phase transition from metastable 1T′ to stable 2H phase in MoS_2_ [39]. Laser irradiation could also induce a phase transition from a stable phase to a metastable one by producing defects in the lattice. Cho et al. successfully converted the 2H-MoTe_2_ into the 1T′-MoTe_2_ locally by laser irradiation and constructed 2H-1T′ heterojunctions, which are stable up to 300 °C [54]. The laser irradiation first thinned the MoTe_2_ film and then induced Te vacancies in the irradiated region, leading to a phase transition (Figure 7c).

Thermal annealing is the most common way to realize phase transformation for various materials. In most cases, thermal annealing could induce or accelerate the conversion of a metastable phase to a stable one by providing energy or healing defects. This can explain the fact that MoTe_2_ exhibited a 1T′ phase at the initial CVD growth stage but gradually changed from a 1T′ phase to a 2H phase with growth over time [23,91]. Based on this, Xu et al. designed a method to prepare large-area single-crystal 2H-MoTe_2_ films from polycrystalline 1T′-MoTe_2_ ones [92]. They put a small piece of single-crystal 2H-MoTe_2_ on a 1T′-MoTe_2_ wafer (with a thickness of 10 nm) as a seed, then deposited a layer of dense Al_2_O_3_ with a thickness of 30 nm on their surface to isolate them from the environment, and finally opened a small hole in the seed region as the channel to transport Te atoms when the wafer was annealing in a Te atmosphere (Figure 7d). When annealing, the Te atoms in the atmosphere diffuse into 1T′ MoTe_2_ via the small hole and heal the Te vacancies in 1T′ MoTe_2_, leading to the phase transition to the 2H phase. The seed can guarantee the phase transition starts in the seed region and the formed 2H phase grows outward until the whole 1T′-MoTe_2_ wafer transforms into the 2H phase. This smart experiment design provides new ideas for the controllable synthesis of large-sized single-crystal phase-change materials.

## 5. Conclusions

In this review, we first discussed the two kinds of phase transformation mechanisms, electronic doping and mechanical shift-induced phase transition, in MoX_2_ nanomaterials and, based on them, presented various phase regulation techniques involving both direct synthesis methods and post treatments. On the basis of our understanding, the advantages and disadvantages of different techniques are summarized: (i) The direct synthesis methods, including CVD and wet-chemical synthesis, as well as ion intercalation, are more readily utilized for large-scale production, and the CVD method can produce large-size TMDC films (monolayer or multilayer), while the others are more suitable to synthesize small-sized ones but with a variety of morphologies, such as nanosheets, nanoribbons, and nanospheres. (ii) The post treatments, including doping, plasma bombardment, and thermal annealing, could process TMDC nanomaterials with many morphologies and are suitable for large-batch production but could not guarantee the phase purity of the products. (iii) The post treatments, including electric field, e-beam irradiation, and laser irradiation, could have accurate control over the phase transformation by choosing proper conditions, but their implements are less convenient. Through the appropriate phase regulation method, the controllable preparation of MoX_2_ nanomaterials with the desired phase structure for specific applications could be accomplished, which may further expand and broaden their future applications.
